# Evaluating the therapeutic effect of LIPUS in the early stage of traumatic brain injury using FA and T2^*^ in rats

**DOI:** 10.18632/aging.206060

**Published:** 2024-08-12

**Authors:** Dan Du, Tao Zheng, Zhanqiu Wang, Yansheng Chen, Shuo Wu, Linsha Yang, Jiabin Lu, Lanxiang Liu

**Affiliations:** 1 Department of Magnetic Resonance Imaging, Qinhuangdao Municipal No. 1 Hospital, Qinhuangdao, China; 2 Department of Radiology, Peking University Third Hospital, Beijing, China; 3 Institute of Medical Technology, Peking University Health Science Center, Beijing, China

**Keywords:** traumatic brain injury, LIPUS, therapy, FA

## Abstract

To evaluate the protective effect of LIPUS at the early stage of brain trauma in rats, 45 rats were randomly divided into 3 groups: sham (*n* = 15), TBI (*n* = 15) and LIPUS treatment groups (*n* = 15). Ipsilateral and contralateral cortical and thalamic parameters obtained by diffusion tensor imaging (DTI) and fast low-angle shot magnetic resonance imaging (FLASH-MRI) were measured at different times after trauma. For fractional anisotropy (FA) and T2^*^ values, two-way repeated measures ANOVA with Tukey’s post hoc was used for intergroup comparisons. With observation time prolonged, the FA values of the ipsilateral cortex in the TBI group gradually increased and were significantly higher than those in the LIPUS treatment group on Day 7 (adjusted *P* = 0.0067). FA values in the contralateral cortex decreased at this time and were significantly lower than those in the LIPUS treatment group (adjusted *P* = 0.0192). Meanwhile, compared with LIPUS group, FA values were significantly higher in the injured thalamus (adjusted *P* = 0.0025). Combined with correlation analysis, FA values were positively correlated with neuronal damage (*P* = 0.0148, r^2^ = 0.895). At 7 days after trauma, T2^*^ values in the ipsilateral cortex of the TBI group were significantly lower. After analysis of ferritin content and correlation, we found that T2^*^ values were negatively correlated with ferritin (*P* = 0.0259, r^2^ = −0.849). By measuring post-traumatic changes in FA and T2^*^ values, it is possible to demonstrate a neuronal protective effect of LIPUS in the early phase of TBI rats and promote brain rehabilitation.

## INTRODUCTION

Traumatic brain injury (TBI) is an important clinical and health-related issue that is prevalent globally, with high morbidity and mortality [1]. It ranges from mild concussion to severe traumatic injuries with neurological sequelae, including cognitive impairment and/or behavioral changes. Currently, the treatment of TBI primarily involves drug therapy. However, not only is their efficacy often restricted by the blood–brain barrier (BBB), but some drugs (e.g., mannitol) may also lead to severe adverse events associated with hepatotoxicity and nephrotoxicity. Long-term functional recovery after central nervous system injury is limited, and currently, no effective treatment is known [2–4]. Others have shown that brain neurons die rapidly within a week after cortical injury, leaving only one-third of the total normal neurons [5, 6]. Thus, establishing a brain protective mechanism in the early stage of trauma is particularly important for treatment, prognosis, and evaluation.

Conventional computed tomography (CT) and magnetic resonance imaging (MRI) examinations can only reflect macrostructural changes after brain trauma but not microstructural changes such as microhemorrhage or axonal injury. This leads to underestimation of the degree of injury. With the emergence of fiber imaging tools, diffusion tensor imaging (DTI) can help visualize the microstructure of the nervous system in a noninvasive manner. This method helps detect the degree of brain edema [7], axonal injury, and glial hyperplasia [8, 9] based on the diffusion of water molecules. Some studies have shown that DTI can sensitively detect mild microstructural changes that exist widely in the whole white matter and gray matter of the brain in the acute stage of brain trauma. Also, DTI can detect progressive secondary tissue damage in the white matter and gray matter [10] in the subacute phase. Fractional anisotropy (FA) is a quantitative indicator of axon-related microstructural changes in DTI, which can be used to evaluate the degree of TBI [11, 12].

Iron imbalance in the brain can result in severe neurodegenerative diseases, including Parkinson’s disease. Iron is often considered a potential mediator of secondary injury after TBI and may contribute to the progression of TBI because it can form free radicals and induce oxidative stress [13]. Because iron is a strong paramagnetic substance that can significantly reduce the transverse relaxation time of the tissue, it lowers signal intensity on T2-weighted images, T2 values, and T2^*^ values. Therefore, T2, T2^*^, and other mapping techniques can be used to indirectly evaluate iron content after brain trauma [14].

Transcranial ultrasound stimulation with high spatial resolution can stimulate specific functional areas of the brain. It is a new noninvasive and effective method of brain stimulation and has shown a unique advantage in treating brain tumors, mental illness and brain degenerative diseases [15, 16]. Using the gadolinium-diethylenetriamine penta-acetic acid (Gd-DTPA) tracer tool to detect the extracellular parameters of TBI tissue, researchers have found that low-intensity pulsed ultrasound (LIPUS) stimulation can significantly expand the brain extracellular space, improve inter-tissue fluid drainage, reduce neuronal and glial cell edema, and promote brain rehabilitation after TBI [17]. In our previous study, we demonstrated that LIPUS can stimulate complete cerebral circulation, increase the number of brain-derived neurotrophic factor (BDNF)-positive cells, and significantly improve the expression of endogenous BDNF in the damaged cortex and thalamus [18].

In the present study, we investigated the protective effect of LIPUS at the early stage of brain trauma in rats using DTI, fast low-angle shot magnetic resonance imaging (FLASH-MRI), and performing immunohistochemical analysis to associate the imaging findings with tissue alterations at the cellular level.

## METHODS

### Animals and groups

In total, 45 Sprague Dawley rats (male; average age, 2 months; average weight, 250 g, provided by Beijing HFK Bioscience, China) were included. The animals were maintained at a temperature of 20–22°C and 60% air humidity, with unlimited access to water and food. The rats were intraperitoneally injected with ketamine/xylazine (100/10 mg/kg) before the operation. These experiments and conditions complied with international animal protection ethics regulations and laws and were approved by the Medical Ethics Committee and Animal Care Committee of the First Hospital of Qinhuangdao, China (No. 20140018).

**Figure 1:**
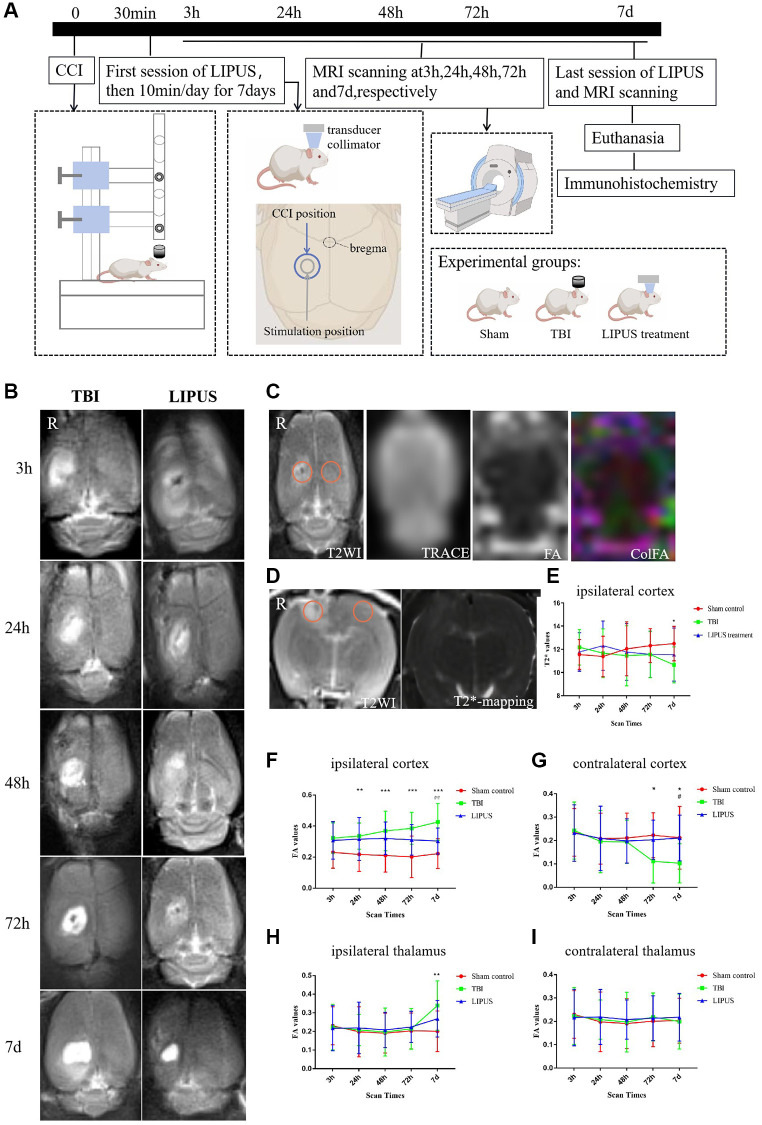
(**A**) Experimental design. Using the CCI surgical operation as the zero point, LIPUS treatment was administered 30 minutes after trauma and then for 10 minutes at a fixed time every day for 7 days. In the schematic diagram of rat skull and brain shown in the figure, the blue circle represents the CCI location, and the gray circle represents the LIPUS treatment location. MRI scans were performed at 3 h, 24 h, 48 h, 72 h, and 7d post-injury. After the last LIPUS treatment and MRI scan, the rats were sacrificed, and their brains were used for immunohistochemistry. The experiment was divided into three groups: Sham, TBI, and LIPUS treatment group. (**B**) Transverse imaging of rat brain at different time points, represents the degree of brain injury in TBI and LIPUS groups. The damaged area is a high signal area of the right cortex (above the hippocampus). As shown, the area of high signal area on tra-T2WI is smaller in the LIPUS treatment group compared to the TBI. (**C**) ROI selection method for quantitative measurement of cortical FA values. Freehand ROIs were carefully matched with cor-T2WI images as reference images. The FA value of the bilateral thalamus was measured by the same method. (**D**) ROI selection method for quantitative measurement of cortical T2^*^ values. Freehand ROIs were carefully matched with tra-T2WI images as reference images. (**E**) Trends of T2^*^ values of TBI, LIPUS treatment and Sham Control groups in the injured ipsilateral cortex. The results showed that on the 7th day after trauma, the T2^*^ values were decreased in the TBI group and significantly different from the Sham group. Two-way repeated measures ANOVA, followed by Tukey’s post hoc. ^*^P < 0.05, Sham control vs. TBI. Data are Mean ± SD. (**F**, **G**) Trends of FA values of TBI, LIPUS treatment and Sham groups in the injured ipsilateral cortex (**F**) and the contralateral cortex (**G**). FA values in the TBI group showed a gradual increase, reaching a peak at 7d, while FA values in the LIPUS treatment group changed relatively flat (**F**). What’s more, FA values reached the lowest on the 7th day in the contralateral cortex (**G**). Two-way repeated measures ANOVA, followed by Tukey’s post hoc. ^**^P < 0.01, ^***^P < 0.001, Sham control vs. TBI. ^#^*P* < 0.05, TBI vs. LIPUS treatment. Data are Mean ± SD. (**H**, **I**) Trends of FA values of TBI, LIPUS treatment and Sham groups in the injured ipsilateral thalamus (**H**) and the contralateral thalamus (**I**). On the 7th day, there was a significant difference between the sham and TBI groups in the injured ipsilateral thalamus (**H**), while no significant difference among groups in the contralateral thalamus (**I**). Two-way repeated measures ANOVA, followed by Tukey’s post hoc. ^**^P < 0.01, Sham vs. TBI. Data are Mean ± SD.

TBI models were developed by performing a Controlled Cortical Impact (CCI) operation. The animals were randomly categorized into three groups (15 rats/group): Sham operation control group (Sham group), brain trauma group (TBI group), and LIPUS treatment group. The rats in the TBI group underwent only the CCI operation, and those in the sham group underwent a scalp incision and skull drilling without the CCI operation. Rats in the LIPUS groups underwent the CCI operation followed by LIPUS treatment, more experimental design reference can be found in Figure 1A.

### CCI operation

The ECCI-6.3 device (Custom Design and Fabrication, USA) was used to construct CCI models. The following parameters were used to induce a moderate CCI through the impact device: the weight of the impact hammer, 21 grams; the diameter of the impact rod, 4.6 mm; the length of the impact tube, 26 cm; and finally, the distance from the lower edge of the catheter 4 mm [19]. The experimental animals were given humane care in accordance with the 3R principle; the rats were weighed before any aseptic procedure, and ketamine/xylazine (100/10 mg/kg) was injected intraperitoneally for anesthesia. Subsequently, the rat was placed on a fixed plate, and its head was fixed; the hair on the top of the head was removed, the skin was disinfected with 75% alcohol, and a 1.5–2 cm incision was made at the center of the area. The skin was peeled off to expose the parietal bone, and the periosteum of the right parietal bone was peeled off. A 5 mm diameter bone window was ground 2.5 mm near the midline and 1.5 mm from bregma to ensure intact dura mater. After the percussion device was disinfected, the dura mater was flushed with the lower end of the catheter and close to the impact rod, ensuring that the catheter was vertical and that the mouse brain was not deflected, allowing the impact hammer to fall freely. After the impact, the impact rod was immediately lifted. After cleaning the surgical area, an intermittent suture was performed. Later, the rats were placed on a warming pad; after they showed physical activity, they were moved back to the cage.

### LIPUS treatment

Thirty minutes after inducing CCI, LIPUS stimulation was administered for 10 minutes once a day for 1 week. The anesthetized rats were placed on the rat brain stereotaxic instrument, and the V301-SU ultrasonic transducer (Olympus, Japan) was fixed on the stereotaxic instrument bracket. The ultrasonic transducer was manually adjusted such that it aimed at the part of the rat’s head that needed to be stimulated. Subsequently, two signal generators (AFG3022C; Tektronix, USA) received the pulse sequence signal. After the signals were amplified using an E&I240 L power amplifier (ENI, USA), the ultrasonic transducer was excited to send out ultrasound. Using a collimator (diameter, 10 mm), ultrasound was administered to the focus area. The collimator and the rat head were coupled with a medical ultrasonic coupling solution. The pulse ultrasound sequence parameters and stimulation scheme used for the treatment were as follows: fundamental frequency (FF) = 500 kHz, pulsed repetition frequency (PRF) = 1 kHz, tone-burst duration (TBD) = 0.5 ms, stimulation duration (SD) = 400 ms. The total stimulation duration was 10 min with 200 trials, and the duration of each trial was 3 s.

### MR image acquisition and postprocessing

The time of model preparation was noted as 0 h, and the model was scanned by Siemens Verio 3.0T with a 4-channel, 50 mm diameter phased array animal coil (part number: 10-F04885, Shenzhen RF Tech Co., Ltd., China) MRI 3 h, 24 h, 48 h, 72 h, and 7 d post-operation. The sequences were as follows:

T2WI (3 min 18 s): repetition number (TR) = 4000 ms, echo time (TE) = 113 ms, average = 6, slice thickness = 2.0 mm, slice number = 10, voxel size = 0.3 × 0.3 × 2.0 mm, field of view (FOV) = 65 × 65 mm, and flip angle = 150°.

Resolve DTI (17 min 12 s): For the MR parameters, slices were aligned parallel to the anterior/posterior line with the following settings: TR = 2000 ms, TE = 100 ms, flip angle = 150°, FOV = 124 × 124 mm, voxel size = 2.3 × 2.3 × 2.0 mm, data matrix = 64 × 64, slice thickness = 2 mm, slice number = 10, diffusion directions = 20, and b-values = 0, 1000 s/mm^2^. A multisegmented k-space filling technique was used to reduce the echo spacing of two adjacent echoes in this sequence.

FLASH (17 min 30 s): TR = 4000 ms, TE = 64/96/181 ms, average = 6, contrasts = 3, slice thickness = 2.5 mm, slice number = 5, FOV = 65 × 65 × 84.4 mm, voxel size = 1.0 × 1.0 × 2.5 mm, and flip angle = 20°.

Imaging analysis was carried out using prototype software on a workstation (Siemens Verio 3.0 T MR Leonardo 3682), especially the image correction of DTI to correct the image distortion caused by slight head movement and uneven magnetic field of rats. FA and T2^*^ maps of rat brains were compared with transition T2WI (Figure 1B) and coronal T2WI slices, respectively. One radiologist with 10 years of experience in neural MRI, who was blinded to the experimental grouping, placed circular regions of interest (ROIs) measuring 0.30–0.60 cm^2^ in the center of the injured ipsilateral, contralateral mirrored cortex and both sides of the thalamus in the TBI and LIPUS groups (Figure 1C, 1D). To increase the accuracy of the measured values, freehand ROIs were carefully matched with cT2WI and tT2WI images as reference images and then copied to corresponding parameter maps. The final values for the injured ipsilateral cortex, contralateral mirrored cortex and both sides of the thalamus were calculated from the mean parameters of the ROI. For the sham group, the same region of the ROI was selected.

### HE staining

After the sections were dewaxed in xylene, dehydrated with fractional alcohol, stained with hematoxylin for 1–2 minutes, rinsed, eosin reverse dyed for 5 minutes, rinsed again, dehydrated with fractional alcohol, and covered with a neutral mounting medium. The sample is then viewed under a microscope, photographed and archived. Each stained section was examined using an optical microscope by a member of the research team with more than 10 years of experience.

### Immunohistochemical staining

At 7 days after trauma, 5 rats in the TBI group and LIPUS group were randomly selected for Nissl staining, and 3 rats in the sham group were selected. Ferritin staining was performed in the same manner.

### Nissl staining

The toluidine blue solution used for Nissl staining was prepared as follows: 5 g toluidine blue (180502, Shanghai Regal Biology Technology Co., Ltd., China) and 5 g sodium tetraborate (C10054060, Macklin Lab, China) were dissolved in 500 mL anhydrous ethanol, stirred evenly, and stored away from direct light at room temperature (RT, 23 ± 1°C) for 48 h. After air-drying for 1 h, the slices were then immersed in the configured toluidine blue solution for 30 min and heated at a constant temperature of 60°C in a water bath. The slices were then soaked in distilled water for 2 min, dehydrated with 70% and 100% alcohol for 2 min and 1 min, respectively, and finally soaked for 1 min each in xylene I and xylene II (20180326, Tianjin Kaitong Chemical Reagent Co., Ltd., China). After staining, the slices were sealed with neutral gum and completely dried. Nissl bodies were observed under an optical microscope. The CAM-MS software program (CNC Software Inc., USA) was used to acquire pictures on the computer, and ImageJ was used to calculate the Nissl body loss rate as the rate of Nissl body loss = (loss of Nissl body/total number of Nissl bodies) × 100 [20].

### Ferritin staining

Brain tissues fixed in 10% formalin buffer were embedded in paraffin and sliced into 4-μm-thick sections. The brain slices were rehydrated in PBS for 10 minutes and then covered with blocking buffers (0.5% Triton X-100 and 5% BSA in PBS) for 1 hour. The slices were then incubated with an anti-ferritin H-chain antibody (EPR18878, Abcam, China) (1:500 dilution) at 4°C overnight. After washing with PBS, IgG-HRP (GB23302, Servicebio, China) (1:200 dilution) was incubated at room temperature for 1 hour. Diaminobenzidine (DAB) was used as a chromogen and hematoxylin was prepared for counterstaining. Six views were chosen randomly from each section and examined using an optical microscope (400×). The average number of ferritin+ cells in 6 sections was calculated and compared across groups. After immunohistochemical staining, tissue sections were observed under a microscope by two experienced researchers who were blinded to any experimental conditions.

### Statistical analysis

Statistical tests were conducted using GraphPad Prism v7.00 (USA). The values are expressed as the mean ± SD. The Shapiro–Wilk test was used to confirm the normality of all data. FA and T2^*^ values were compared by a two-way analysis of variance for repeated-measures ANOVA and Tukey’s test. To compare the rates of Nissl body loss and the number of Ferritin H-chain positive cells, the one-way ANOVA was used. The threshold of statistical significance was *P* < 0.05.

## RESULTS

### FA values of the ipsilateral cortex

Compared with the sham group, the FA values were higher in the TBI group and the LIPUS treatment group at 3 h after trauma (TBI group vs. LIPUS treatment group vs. sham group, 0.323 ± 0.098 vs. 0.308 ± 0.121 vs. 0.231 ± 0.102) but not significantly different from each group (adjusted *P* = 0.0578, adjusted *P* = 0.1340, respectively). With the prolongation of observation time, FA values in the TBI group showed a gradual increase, reaching a peak at 7 d, while FA values in the LIPUS treatment group changed relatively flat (Figure 1F).

### FA value of the contralateral cortex

In the TBI group, FA decreased from 3 h after trauma and reached the lowest value on the 7th day, while the dynamic curves of the LIPUS-treated and sham rats were relatively similar. At the 7 d time points, FA was markedly different between the TBI and sham groups (adjusted *P* = 0.0144 and 0.0178, respectively) (Figure 1G).

### FA value of the ipsilateral thalamus

In the TBI group, FA increased from 72 h after trauma; the value was significantly different from that of the sham rats on the 7th day (adjusted *P* = 0.0025), and the FA value in the LIPUS group increased less than that in the TBI group at 72 h and was also not significantly different from those of the sham and TBI groups (adjusted *P* = 0.2319, adjusted *P* = 0.1942) (Figure 1H).

### FA value of the contralateral thalamus

The change curves of the TBI, LIPUS treatment, and sham groups showed similar trends, and there was no significant difference (Figure 1I).

### T2^*^ value of the ipsilateral cortex

The change curves of the TBI, LIPUS treatment, and sham groups showed similar trends before 72 h, but on the 7th day, there was a significant difference between the sham and TBI groups (adjusted *P* = 0.0283) (Figure 1E).

### HE staining

**Figure 2:**
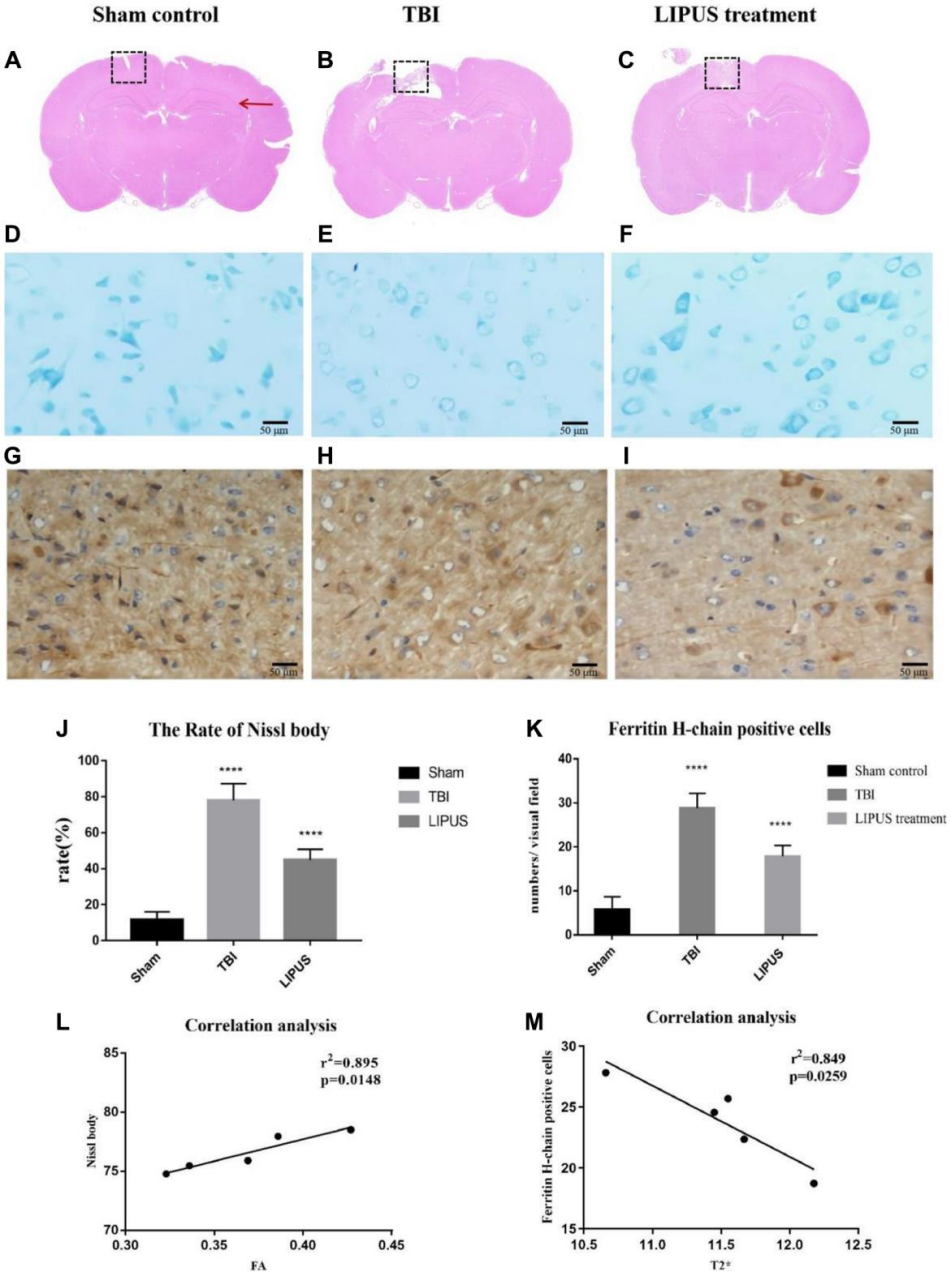
(**A**–**C**) Representative HE staining images of different groups are shown. The red arrows indicate the location of the hippocampus, and the dashed boxes represent the site of cortical injury on the right side. It can be observed that there was significant cavity formation in the TBI group, and the cavity area decreased after LIPUS treatment. (**D**–**F**) Histopathology of Nissl staining. Compared with the Sham control group, the neurons in the TBI group showed obvious edema, fewer cells, sparse arrangement, reduced Nissl bodies in the cytoplasm, and some cells were stained light blue. In the LIPUS treatment group, the nerve cells in the injured area were arranged densely and neatly, and the Nissl bodies were abundant in the cytoplasm. The Nissl corpuscles in the cerebral cortex were dark blue, the nucleus was light blue, and the background was light blue. Bar, 50 μm. (**G**–**I**) Immunohistochemical staining of Ferritin-H-chain in the ipsilateral cortex 7th day after TBI. Brown cells represented positive for ferritin-H-chain, which were mainly located in neuron-like cells and occasionally in glia-like cells. The number of positive cells in TBI group and LIPUS group was significantly higher than that in Sham group. Obvious brown staining was observed in the cytoplasm in TBI group, and the staining in LIPUS group was lighter than that in TBI group. (**J**) The rate of Nissl body in the LIPUS group was obviously lower than TBI group. (**K**) The number of positive cells in TBI group and LIPUS group was significantly higher than that in Sham group. (**L**, **M**) Correlation analysis. In the TBI group, FA values were positively correlated with neuronal damage and T2^*^ values were negatively correlated with ferritin. Data are Mean ± SD. One way-ANOVA, ^****^P < 0.0001.

HE staining was used to observe the pathological changes of the injured site. The images showed that brain tissue in the sham group remained intact, while cortical tissue in the other two groups was observed with varying degrees of tissue defects. There was significant cavity formation in the TBI group, and the cavity area decreased after LIPUS treatment (Figure 2A–2C).

### Nissl staining

From morphological observation, Nissl bodies were deeply stained and stained uniformly in the sham group, and their shape and size were basically the same, while Nissl bodies in the TBI and LIPUS treatment groups were unevenly stained, with light cytoplasmic staining and inconsistent shape at 3 h, and some of them showed cavitation as time extended, with the TBI group being the most prominent (Figure 2D–2F). From the semiquantitative measurements, the loss rate of Nissl bodies was higher in both the TBI group and the LIPUS treatment group than in the sham group, and in descending order of loss rate, it was TBI group (77.57 ± 5.21%) > LIPUS treatment group (46.49 ± 4.35%) > sham group (11.18 ± 6.54%~12.43 ± 8.74%) (Figure 2J).

### Ferritin staining

Obvious brown staining was observed in the cytoplasm of TBI rats, and the staining intensity in the LIPUS treatment group was lighter than that in the TBI group (Figure 2G–2I). Fe-H staining in the ipsilateral cortex on the 7th day after TBI revealed a small number of ferritin+ cells in the cerebral cortex in the sham group (5.56 ± 3.0/visual field). The TBI and LIPUS treatment groups had significantly higher numbers of ferritin+ cells than the sham group (22.38 ± 3.12/visual field and 18.38 ± 1.98/visual field, respectively) (Figure 2K).

### Correlation analysis

In the TBI group, FA values were positively correlated with neuronal damage (*P* = 0.0148, r^2^ = 0.895, (Figure 2L), and T2^*^ values were negatively correlated with ferritin (*P* = 0.0259, r^2^ = 0.849, Figure 2M).

## DISCUSSION

Our study found that the FA value of the cortex on the injured side of TBI rats was dramatically higher than that of sham rats at 3 h after trauma and increased continuously with time, reaching a peak on the 7th day. The FA value of LIPUS-treated rats was also markedly higher than that of sham rats at 3 h after trauma but did not show an upward trend with the progression of time, instead remaining similar to that of the sham group. Ling et al. [21] reported an increase in FA values in the cortical and subcortical areas of patients with brain trauma for the first time. They suggested that cytotoxic edema caused an increase in FA values in the acute phase (3 h), and the reaction of glial cells and the formation of glial scars destroyed the surrounding cellular environment, leading to a continued increase in FA values in the subacute phase [22]. Similarly, Vasiukova et al. suggested an increase in FA values due to the entry of water into the axonal interstitium during the acute phase, resulting in axonal swelling and the presence of microstructural damage [23]. Studies have shown that ultrasound increases unidirectional water transport in chondrocytes cultured *in vitro* [24]. Yoon [25] and other research groups have also confirmed that ultrasound can increase water transport through the BBB, leading to increased water transport in the blood vessels, which would help to reduce brain edema. However, extensive protein exudation occurs 4–6 h after the destruction of the BBB [26]. Therefore, the application of ultrasonic stimulation in the early stage of brain trauma can not only effectively promote the repair of the BBB but also prevent its progressive damage. Nissl staining showed that the loss rate of Nissl bodies in TBI rats was much higher than that in LIPUS-treated rats. With the progression of time, the loss of Nissl bodies in LIPUS-treated rats tended to remain stable. Moreover, correlation analysis showed that FA values were positively correlated with neuronal damage in the TBI group, which further proved that LIPUS could reduce trauma-induced brain edema and damage to nerve cells.

In this study, we also found that the trend of change in FA values in the contralateral cortex of TBI rats contrasted with that in the ipsilateral cortex, and the FA value was lowest on the 7th day. This could be because delayed neuronal death, decreased nerve regeneration [27], Waller degeneration, axon injury, and myelin degeneration all caused changes in the contralateral brain tissue after trauma. When the contralateral cortical white matter was damaged, the dispersion became asymmetrical, resulting in a decrease in FA [28, 29]. In the LIPUS treatment group, the FA value of the contralateral cortex did not decrease significantly; in fact, the FA value was mostly maintained at the baseline level, which indicated that LIPUS inhibited delayed neuronal death, inhibited the increase in cell membrane permeability, and improved the extracellular space in the brain.

TBI can not only cause local and direct damage but can also cause indirect damage to distal brain areas such as the thalamus and corpus callosum [30]. Our study found that the FA value of the thalamus on the injured side in the TBI group began to increase at 72 h and peaked on the 7th day, which further confirms the existence of persistent injury after brain trauma. Similarly, the results published by Soni et al. and other research groups have also shown that the expression of GFAP-positive cells in the injured thalamus reaches a peak on the 7th day. Axons in the white matter are easily damaged after brain injury. It was suggested that there was persistent damage in the thalamus even though it was far from the site of injury [31]. The activation of thalamic microglia is closely related to injury of the white matter bundle in the thalamic cortex [32]. Owing to the high density of connections between the thalamus and the injured axons, microglia can be seen not only in the retrograde projection area but also in the damaged neurons and anterograde areas. The activation of microglia causes a series of inflammatory immune responses, aggravates the swelling of brain tissue after injury, increases the permeability of the cell membrane, decreases the density of axons, and increases the tissue composition of the extracellular space [33], thus affecting the index of anisotropy. However, the increased FA values of the thalamus on the injured side of LIPUS-treated rats were not obvious compared to TBI rats on the 7th day, which further proves that LIPUS could reduce axonal injury caused by brain trauma and play a protective role in the thalamus.

Cerebral microhemorrhage is a common feature of brain trauma and several neurodegenerative diseases and is observed in both acute and chronic brain injury phases during experimental modeling of single and repeated brain injury [34]. Intracranial hemorrhage and the destruction of the BBB after craniocerebral injury can promote the release of heme, which may lead to iron overload [35]. Similar to previous studies, which detected an increase in iron concentration at the impact site 3 days after trauma, with the increase lasting for 28 days [36, 37], our study found that on the 7th day after trauma, the T2WI signal and T2^*^ value of the injured cortex in TBI rats decreased, while the T2^*^ value in the LIPUS treatment group decreased slightly. However, they used the laser ablation-inductively coupled plasma–mass spectrometry imaging method, which can sensitively reflect the metabolism of metals in the brain. As one of the most important iron storage proteins, ferritin may regulate iron-induced cytotoxicity, lipid peroxidation, and the formation of free radicals. Ferritin contains 2 subunits, the light ferritin (L) chain and the heavy ferritin (H) chain. The ferritin H chain is expressed primarily in neuron-like cells and is responsible for iron uptake and reuse, while the L chain is involved in long-term iron storage. Therefore, the changes in H-chain ferritin were studied [38]. The results showed that there were a small number of Ferritin+ cells in the cerebral cortex of sham rats, higher numbers in the LIPUS group, and even higher numbers in the TBI group. To further demonstrate the relationship between the T2^*^ value and ferritin, by correlation analysis, we found that H-chain ferritin-positive cells were negatively correlated with the T2^*^ value. Based on these results, we infer that LIPUS could reduce iron deposition in the brain after trauma and regulate the iron imbalance in the brain. This lays a foundation for us to further study the effect of LIPUS on iron metabolism in the brain.

In summary, by measuring the changes in FA and T2^*^ values after trauma, it was demonstrated that the application of LIPUS treatment in the early stage of brain trauma could protect against neuronal damage, while maybe participate in the regulation of iron homeostasis in the course of long-term disease progression, then improve prognosis and promote brain rehabilitation in rats with TBI.

There are some limitations to this study. First, although the MR equipment included special rat coils used for image acquisition, higher quality images are not available. Secondly, the single method does not give full play to the advantages of multi-sequence and multi-parameter magnetic resonance. Therefore, the therapeutic effect of LIPUS should be evaluated from a multi-modal perspective, and new techniques, such as Diffusion kurtosis imaging (DKI), quantitative susceptibility mapping (QSM), should be added to seek favorable evidence. Third, we did not include the condition of a drug treatment group and/or the combination of LIPUS and drug treatment group, owing to the experimental conditions. Further studies are needed to combine LIPUS with multiple therapeutic agents and elucidate the potential role of both physiotherapy and pharmacotherapy in the treatment of TBI.
